# Reducing Psychological Impacts on Children with Chronic Disease via Family Empowerment: A Scoping Review

**DOI:** 10.3390/healthcare10102034

**Published:** 2022-10-14

**Authors:** Ai Mardhiyah, Santhna Letcmi Panduragan, Henny Suzana Mediani

**Affiliations:** 1 Faculty of Health Science, Lincoln University College, Petaling Jaya 47301, Malaysia; 2 Department of Pediatric Nursing, Faculty of Nursing, Universitas Padjadjaran, Bandung 40132, Indonesia; 3 Faculty of Nursing, Lincoln University College, Petaling Jaya 47301, Malaysia

**Keywords:** children, chronic disease, family empowerment

## Abstract

Chronic diseases cause physical and psychological impacts on sufferers. In dealing with illness, the family is not involved in the treatment of chronic diseases. Children also do not receive support from their families in dealing with their illness. Family empowerment is an important thing to implement in treating children with chronic diseases. The purpose of this study was to explore family empowerment interventions as potential methods to reduce the impact of chronic disease. This study used the scoping review method. A literature review was conducted via CINAHL, PubMed, and ProQuest databases. The keywords used in English were “family empowerment OR family center empowerment” AND “child OR children” AND “chronic disease”. The criteria for articles in this study were full text, free access, randomized control trial or quasi-experiment research design, English language, population and samples of chronic disease, and the publication period of the last 10 years (2013–2022). We found nine articles that used a family empowerment intervention in an effort to reduce the impact of chronic disease on children. Most of the study designs were randomized control trial and quasi-experiment. Some of the benefits of family empowerment interventions were quality of life, family care, and self-ability. The interventions helped the families to be empowered and actively participate in caring for children with chronic diseases. There were nine articles that discussed family empowerment interventions that have an impact in dealing with the impact of chronic disease on children, namely improving quality of life, family care, and self-ability.

## 1. Introduction

Chronic disease is a non-communicable disease that can cause death and has a long duration, and develops slowly. The causes of chronic disease are a combination of physiological, genetic, behavioral, and environmental factors [1]. The WHO (2018) has stated that there will be an epidemiological transition from infectious diseases to non-communicable diseases in 2030 [2]. Several types of chronic diseases cause limitations and disabilities in sufferers [3]. Chronic disease can lead to hospitalization due to limitations in daily activities [4]. Children who are hospitalized today are experiencing more serious and complex problems, and often children who experience long-term hospitalization are at high risk of experiencing disturbances in child development [5].

Chronic diseases include hypertension, diabetes mellitus, cancer, kidney failure, and so on. Chronic diseases kill more than 36 million people every year. Deaths due to cardiovascular disease are mostly caused by PTMs and affect as many as 17.3 million people per year, followed by cancer (7.6 million), respiratory disease (4.2 million), and DM (1.3 million). These four groups of disease types cause about 80% of all PTM deaths [6]. Chronic kidney disease affects an average of 10% of the population worldwide—the Indonesian Renal Registry (IRR) noted that in 2015 there were 30,544 active patients undergoing hemodialysis, most of whom were chronic kidney failure patients [7].

The impacts of chronic disease on development include the presence of motor disorders, speech disorders, personal social disorders, mental development disorders (such as mental retardation), behavioral development disorders (such as hyperactivity), learning disorders, depression, and others [8]. The impact of chronic disease also occurs on the psychological side, namely teenagers feel guilty for their families, but on the other hand, children will demand more attention because they feel helpless [9]. Meanwhile, the psychosocial impacts that occur in children include having a slightly higher risk of experiencing symptoms of depression than healthy children, withdrawing from the environment, and losing hope [10]. Additionally, the psychosocial impacts of chronic illness can be related to educational prospects, fear of limitations provided by the environment, and anxiety about the reactions of others, especially peers, to their illness [11]. Efforts are being made to improve the ability of children in self-care by empowering their families [12]. Family empowerment is a family-based strategic intervention that can increase the role of the family in caring for patients. The purpose of family empowerment is to help families to care for and provide assistance to family members with chronic diseases as an important element of the care process [13]. Nurses also have a role in advocating and encouraging families in the self-care management planning process for patients with chronic diseases [14]. The results of research showed that from 56 family respondents, 67.9% of families who cared for patients had low levels of empowerment due to a lack of information about interventions that could be carried out by families [15]. In addition, Gomes et al., (2017), who evaluated family involvement in controlling DM2 by providing educational interventions, showed that increasing glycemic control reduced HbA1c results in DM2 patients [16].

Family empowerment can optimize the role, function, and support of families in the care of patients with chronic diseases at home. To achieve optimal family empowerment, strong belief is needed to prepare and be able to carry out a thorough evaluation to treat patients [17]. Family empowerment is an important thing to do in order for the family to be able to carry out patient care, so based on the information above, the researchers were interested in conducting a literature study on family empowerment in children with chronic diseases.

## 2. Materials and Methods

### 2.1. Study Design

This study was designed using Arksey and O’Malley’s scoping review framework. Scoping review is a methodological technique to explore new topics that are currently being developed [18]. This research framework has a wide conceptual range so that it is able to explain various relevant studies [19]. The framework used consists of 5 core stages, namely identification of research questions; identification of relevant study results; study selection; mapping data; and compiling, summarizing, and reporting results [20]. This literature review used the PRISMA Extension for Scoping Reviews (PRISMA-ScR) to identify various studies that discussed interventions to increase resilience in children during the COVID-19 pandemic. The first question to initiate the search process was: what is the effect of family empowerment on children with chronic disease?

### 2.2. Search Strategy

For publication searches, three databases were used: PubMed, CINAHL, and Proquest. The keywords and Boolean operators used were: “family empowerment OR family center empowerment” AND “child OR children” AND “chronic disease”.

### 2.3. Inclusion and Exclusion Criteria

**Figure 1:**
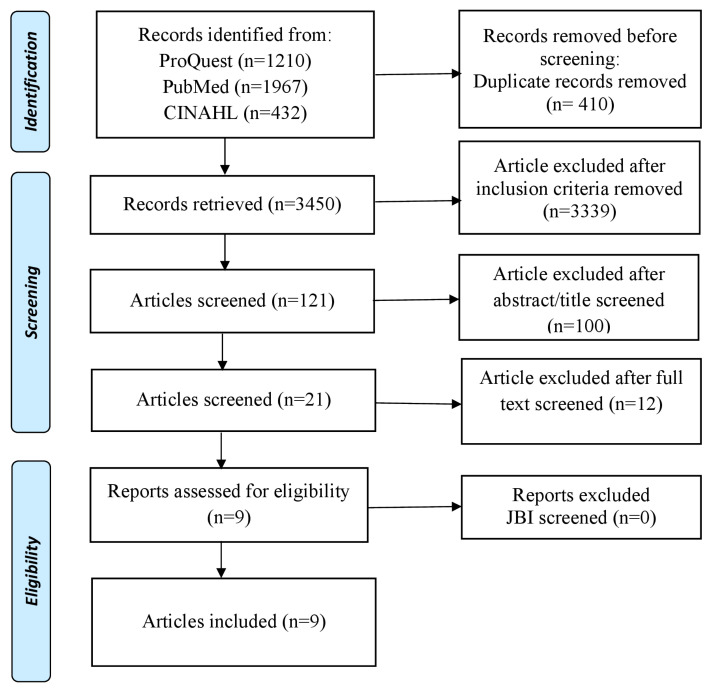
PRISMA Flow Diagram.

This literature review used the PRISMA Extension for Scoping Review (PRISM-ScR), which served to identify various studies that discussed interventions to reduce the impact and behavior of bullying on students in schools (Figure 1). Articles were selected for review based on inclusion and exclusion criteria. The inclusion criteria of this study were that the patient was a student, primary research, there was intervention, the article was original research, used English, full text, and time setting of the last 10 years (2013–2022).

### 2.4. Eligibility Criteria

The criteria in this study based on the PICO criteria framework were:

Patient: children with chronic disease;
Intervention: family empowerment;
Comparison: no comparison;
Outcome: effect, quality of life, family care.

In addition to the eligibility conditions specified above. We considered the criteria chosen by the authors in the primary research results, which used randomized control trial and quasi-experimental design to describe the family empowerment intervention. The articles used were English articles with full text, published within the last 10 years (2013–2022).

### 2.5. Quality Appraisal

Articles were analyzed by authors using the Joanna Briggs Institute (JBI) critical assessment method. JBI critical assessment is a tool to critically assess articles based on the quality, trustworthiness, and results of published articles. The article assessment method uses a scoring consisting of yes, no, unclear, and not applicable. A yes score is given 1 point and other answers are given 0 points. In the article with the randomized control trial design, there were 13 statement points and the quasi-experiment contained 9 statements. Each point was added to determine the eligibility of articles, with a score above 75% as a good standard for review in this study.

### 2.6. Data Collection and Analysis

All authors completed the study selection process and included studies by following the PRISMA flowchart: (1) identifying duplicates, (2) title and abstract filtering, and (3) full text availability. Tabulation method was used to extract data from research results manually. Among the data items searched for were author, country, study design, objectives, interventions, and outcomes.

## 3. Results

In total, 3609 articles were obtained from the search. After removing duplicates from the collected articles, 3450 articles remained. Furthermore, after elimination based on the inclusion criteria, there were 121 articles remaining. Then, after checking the title and abstract, 21 articles were obtained. Following this, we read and analyzed the full text of the articles and, ultimately, 9 articles were included in this study. Articles were analyzed using the JBI Critical Appraisal Tool assessment method with good article standards above 75% based on criteria and topic relevance (Table 1).

There were nine articles that described family empowerment interventions in children with chronic diseases. Researchers identified the five impacts based on the intervention method used. The results of the analysis of the article are presented in tabular form as follows (Table 2):

From the nine articles analyzed, there are family empowerment interventions that can be useful for children, namely increasing quality of life, increasing family care abilities, and increasing self-ability. Based on the results of this scoping review, the following is an explanation of the impact of family empowerment interventions on children with chronic diseases, classified based on the impact.

### 3.1. Quality of Life

Quality of life of children with chronic diseases is important in order to be able to maintain the capability of children to heal. Quality of life needs to be improved in children with chronic diseases because health conditions can be assessed based on physical health, psychological, social relationships and the environment. Shoghi et al. (2019) studied family-focused empowerment in groups, face to face, using a lecturer system, brainstorming, and educational teaching aids, including power point presentations, films, and educational replicas. This training was carried out in four steps. The first step was to increase the level of knowledge, using several teaching aids, namely PowerPoint presentations, models, posters, team teaching, question and answer sessions, lectures, and role plays [21]. The second step was to increase self-efficacy using the demonstration method. During the demonstration, an explanation of the skills required for the measurement of weight and blood pressure was given, and these skills were taught to children through demonstrations. The third step was increased self-esteem through participatory training. In this step, parents were involved in identifying the issues of the chronic disease suffered, namely chronic kidney disease. In this method, children transferred the knowledge gained through the materials used in their respective group discussions and observations in self-efficacy sessions to their parents. The fourth step (process evaluation) had the aim of evaluating the knowledge of parents. At the beginning of each session, the children verbally asked some questions about the material from the previous session. The results showed that family empowerment interventions were able to measure the training and treatment needed and were low-cost and effective techniques. Interventions can also help improve children’s self-efficacy and quality of life in dealing with chronic illness.

Other research has shown that the family-centered empowerment model has four stages that can improve the quality of life of children with chronic diseases [24]. The first stage was to increase knowledge or understanding of the consequences of the severity and sensitivity felt by family members. At this stage, two educational sessions of at least 45 min were held that discuss the physiology of rheumatoid arthritis, symptoms, complications, prognosis, drugs, nutrition, activities, and exercise. After being educated, the topic was explained again in case of ambiguity. Before the next session began, participants were asked again about the understanding of the material previously obtained. The second stage was increasing self-efficacy, with the aim of increasing self-efficacy through increasing the individual’s ability to perform self-care actions. The third stage was known as self-esteem boosting, which was done through training pieces for the participants. The fourth stage of the program was the assessment step. At this stage, evaluation was carried out during the intervention until the training was complete. The assessment was carried out in order to review the steps of the empowerment plan by using questions and answers at the end of each session and at the beginning of the next session.

Another intervention was carried out through four educational sessions held within two weeks to make patients and family members feel positive and increase their level of knowledge [28]. In the first session, the family is taught about problem-solving methods—the patient states the problem and the researcher gives an explanation of the problem. The second phase was validation, in which the family was asked to re-explain. In the third phase (participatory education), the patient was asked to study the pamphlet at home after each session, which was done during the first session and the second phase was continuous. The fourth phase (as an assessment) was carried out in two steps, namely an oral assessment related to the material being taught and written questions.

### 3.2. Family Care

Care for children can be carried out with a focus on empowering families to be able to carry out independent care for children [22]. To study this, an empowerment program was carried out in several sessions. In the first session (40 min), using educational books, researchers spoke face to face with parents about types of cancer in childhood, common signs and symptoms of cancer, diagnostic methods, and treatment programs according to stage and disease conditions of the child. Then, the family was given a book about the material. Then, in the second session, lasting 40 min, the family was asked to discuss with the hospital care team about treatment, assessment, and control of the effects of chemotherapy. Following this, there were other issues to discuss, such as ways to prevent infection, control and relieve pain, vaccinations, improving self-control in dealing with sick children and their problems, as well as ways to support other family members. Then, in the third session, each participant was asked to teach one of his family members who cared for the patient under the supervision of the researcher. In the fourth session, the final evaluation session, the researcher assessed all participants in the intervention group by asking questions about all the content taught and the issues discussed during the second and third sessions. The researcher concluded that things that were still ambiguous.

Another family empowerment intervention that has been studied is the Family Empowerment Program (FEP) [23]. The FEP intervention consists of five stages, namely seeking reality and exploring hopeful beliefs about the illness; considering choices and making decisions related to family problems and needs, children’s emotions and emotional responses, and the impact of the disease on children and their families; developing the ability to manage care by sharing information and experiences and learning from other families about family functions and family management; strengthening self-confidence to master family situations that involve managing the child’s care effectively and being able to participate effectively in activities of daily living; and reflecting and providing feedback on changes in their family’s competence and level of trust.

### 3.3. Self-Ability

Family empowerment interventions are useful for increasing self-efficacy, including increasing self-esteem, self-efficacy, lifestyle, and reducing the length of hospital stay. An intervention that has been carried out to increase self-esteem is the Family-Centered Empowerment Model (FCEM) [25]. The implementation of FCEM consisted of four steps. The first step was to increase knowledge, carried out by means of training sessions with group discussions and educational booklets on topics, namely: (1) physiology, symptoms, complications, and prognostic factors; (2) asthma; (3) nutrition, physical activity, and exercise; and (4) medical care. The second step was to promote self-efficacy through demonstrations and by explaining self-efficacy to families. The third step was to increase self-esteem. In this step, children and parents discussed the problems associated with asthma and were encouraged to take care of it. The fourth step was evaluation, which included the evaluation process of each step that had been carried out. This intervention was able to increase the self-esteem of children with chronic diseases so that they are able to have hope for the healing process of their disease.

Another step in improving self-efficacy is the Family-Centered Empowerment Program (FCEP) [26]. At the initial stage, the family was given knowledge about the disease via a group discussion through three training sessions for 30–45 min, including two group discussion sessions and problem-solving and individual counseling sessions. The second step was to increase self-efficacy by finding solutions and solving the problems discussed in the first step. Then, the third step was to increase participation by sharing the information that had been obtained from the previous two steps. The fourth step was an evaluation program through oral questions. After the intervention, the efficacy of children with chronic diseases increased so that there was hope for the healing process of the disease.

Family empowerment interventions have also been shown to affect the length of care for children with chronic diseases, namely pneumonia in hospitals with the Family Empowerment Model (FEM) [27]. To achieve this, the steps that have been studied include providing health education related to pneumonia using flipcharts and booklets, with a maximum duration of 45 min. The family empowerment module, which contained information about pneumonia and nursing care for children with pneumonia, focused on family, family empowerment, and concepts related to empowerment (motivation, caring, social support, and self-efficacy). This study showed that their lifestyle changed for the better by reducing the risk of aggravating the effects of leukemia on children. The results showed an increase in family satisfaction and empowerment. Another important indicator of the success of FEM is its ability to reduce patient length of stay.

Lifestyle changes are an important thing to achieve in the process of family empowerment. Lifestyle changes that have been studied include self-efficacy, self-esteem, and self-control against leukemia [29]. The steps taken started with group discussion sessions with active patients and family members to find out the impact of the disease. These were followed with disease problem solving sessions that involved family participation to increase self-efficacy, self-esteem, and self-control. Next, was educational participation, encouraging patients to talk to their families about previous learning. Lastly, was an evaluation to confirm the program and determine the follow-up of the program. Implementation of a family-centered empowerment model for pediatric patients with leukemia showed that there are changes in lifestyle for the better to reduce the impact of leukemia on children.

## 4. Discussion

Families have an important role in helping to care for children with chronic diseases. Family participation is not only needed to provide support for the child’s healing, but also involvement in providing care. Therefore, family empowerment is an important factor in improving the healing process of chronic diseases in children [30].

Family empowerment is an intervention with a holistic approach that focuses nursing care on children and families as clients or individuals with biological, psychological, social, and spiritual needs [31,32]. Family empowerment interventions use the freedom of interactions between parents and children, with the aim to create a more flexible space, e.g., chatting in several places, such as the lobby, study room, play room, bedroom, and foyer [33].

Based on the results of the study, most of the literature showed that many family empowerment interventions occurred in developing countries, such as Egypt, Iran, and Indonesia. Although these programs do not only occur in developing countries, the gaps in knowledge around chronic disease are also caused by family income in developing countries [34]. Previous studies have shown that low income causes low family knowledge, meaning that families have not been able to increase their understanding of chronic diseases [35]. Lower incomes also cause families to be less likely to undertake costs related to programs that would help to improve the ability of families to manage children with chronic diseases [34]. Meanwhile, families with high incomes have good knowledge about managing children with chronic diseases [36]. This is because there is a lot of information and services that cost money in managing children with chronic diseases.

Families have an important role in helping care for children with chronic diseases. Family participation is not only needed to provide support for the child’s healing, but also in providing care [37]. Therefore, family empowerment is an important factor in improving the healing process of chronic diseases in children.

Family empowerment interventions provide many benefits, such as increasing confidence and ability to care for children with chronic illnesses. In addition, the family can also reduce the family’s dependence on professional nursing providers so that they can reduce the length of care and reduce the cost of care. In addition, these interventions can improve the mental health of the child, such as quality of life, self-efficacy, self-esteem, social support, self-ability, and lifestyle [24,38,39].

The steps taken in family-centered care are of two types, namely there are four-step and five-step programs. The four-step method used in family empowerment is education to increase knowledge, increasing self-efficacy using the demonstration method, increasing self-esteem through participatory training, and evaluation to measure the success of the activities that have been carried out [37,40]. Meanwhile, the five-step method includes perceiving threat, self-efficacy, educational participation, evaluation, and the final evaluation [25,41].

The principle used in implementing family empowerment is to focus on the ability of families to deal with chronic disease problems in children. Other research has shown that the FCC principle is the empowerment of children and families to find self-strength, grow self-confidence, and make choices and decisions in health [42]. The family empowerment is carried out in the form of increasing knowledge, participation, skills, and creating a conducive environment for the child’s care [43].

The role of parents in family empowerment is in the form of involvement in physical care, namely personal hygiene, temperature measurement, reducing child fever, providing nutrition, and childcare [44,45]. Nurses have a role as educators to increase knowledge and understanding of parents about their child’s care and provide information about every action taken. Health education is given orally, through PowerPoint presentations, and through websites [32,46]. After being given the activity, it is hoped that it will increase the understanding of children and families, as well as the involvement of families and children in treating chronic diseases.

### Limitations

The limitation in this study is that the research design from the literature is limited to RCTs and quasi-experiments, so it cannot comprehensively discuss family empowerment interventions to reduce the psychological impacts on children with chronic diseases. In addition, this study also has limitations, namely the year of publication of the literature was limited to the last 10 years, which causes this study to be unable to discuss interventions outside the specified time. Another limitation is that the database search was limited to three databases, which caused the literature to be less comprehensive because we could not discuss articles that studied the role of family empowerment in reducing the psychological impact on children with chronic diseases from other databases.

## 5. Conclusions

Based on the results of a literature review, there were nine articles that described family empowerment interventions to reduce the impact of chronic disease on children. The benefits of family empowerment interventions are quality of life, family care, and self-ability. The interventions provided focused on helping families to be empowered and actively participate in caring for children with chronic diseases. Family empowerment can be implemented by the family either at home or in the hospital, because it can reduce the length of hospital stay for children with chronic diseases. The implication of this study is that there is a basis for health workers to develop family empowerment intervention methods in reducing the psychological impact on children with chronic diseases. In addition, health services can also provide policies in implementing and promoting family empowerment to reduce the psychological impact on children with chronic diseases. This study can also be the basis for further research to examine the effectiveness of family empowerment in reducing physical and psychological impacts on children with chronic diseases.

## Figures and Tables

**Table 1:** JBI Critical Appraisal Tool.

Author, Published Year	JBI Critical Appraisal Tool	Study Design
[21]	88.8% (8/9)	Quasi-experimental
[22]	88.8% (9/0)	Quasi-experimental
[23]	76.9% (10/13)	Pilot study RCT
[24]	76.9% (10/13)	RCT
[25]	88.8% (8/9)	Quasi-experimental
[26]	84.6% (11/13)	RCT
[27]	77.8% (7/9)	Quasi-experimental
[28]	92.3% (12/13)	RCT
[29]	84.6% (11/13)	RCT

**Table 2:** Extraction data.

No	Author and Year	Outcome	Location	Method	Sample	Intervention	Result
1.	[21]	Self-efficacy and quality of life	Egypt	Quasi-experimental	68 children	Family empowerment model with four steps	The results of the intervention showed that there was a significant increase in children’s self-efficacy and quality of life.
2.	[22]	Burden	Tehran	Quasi-experimental	78 parents of children with cancer	Family-centered empowerment model (FCEM) with four sessions	The results of this study indicated that it was effective in reducing the burden of caring for children with cancer.
3.	[23]	Social support and ability	Buapa University, Muang	Pilot RCT	25 parents with children thalassemia	Family empowerment program with five stages	The results of the intervention showed that there was an increase in social support and care for children.
4.	[24]	Quality of life	Iran	RCT	60 children	Family-centered empowerment model with four stages	The results of the intervention showed that there was a significant improvement in the quality of life of children.
5.	[25]	Self-efficacy and self-esteem	Iran	Quasi-experimental	60 children	Family-centered empowerment model with four steps	The results of the intervention showed that there was a significant increase in self-efficacy and self-esteem in children.
6.	[26]	Self-efficacy	Iran	RCT	70 Children	Family-centered empowerment program with four steps	The results of the intervention showed that there was an increase in self-efficacy in children with thalassemia major.
7.	[27]	Family satisfaction and children’s length of stay in the hospital	Indonesia	Quasi-experimental	83 family–child groups	Family empowerment model with four steps	The results of the intervention showed that there was family satisfaction and empowerment, as well as reducing the length of stay for children.
8.	[28]	Quality of life	Iran	RCT	46 children	Family-centered empowerment model with five steps	The results of this study indicated that the intervention was effective for improving the quality of life in children with leukemia.
9.	[29]	Lifestyle	Iran	RCT	30 children	Family-centered empowerment with five steps	The results of the intervention showed that there was a better modification of the patient’s lifestyle to improve health status.

## Data Availability

Not applicable.
